# Gallin; an antimicrobial peptide member of a new avian defensin family, the ovodefensins, has been subject to recent gene duplication

**DOI:** 10.1186/1471-2172-11-12

**Published:** 2010-03-12

**Authors:** Daoqing Gong, Peter W Wilson, Maureen M Bain, Karina McDade, Jiri Kalina, Virginie Hervé-Grépinet, Yves Nys, Ian C Dunn

**Affiliations:** 1 Roslin Institute and Royal (Dick) School of Veterinary Studies, University of Edinburgh, Roslin, Midlothian EH25 9PS, UK; 2 University of Glasgow, Faculty of Veterinary Medicine, Glasgow, G61 1QH, UK; 3 College of Animal Science and Technology, Yangzhou University, Yangzhou 225009, China; 4 INRA, UR83 Recherches Avicoles, 37380 Nouzilly, France

## Abstract

**Background:**

Egg white must provide nutrients and protection to the developing avian embryo. One way in which this is achieved is an arsenal of antimicrobial proteins and peptides which are essentially extensions of the innate immune system. Gallin is a recently identified member of a family of peptides that are found in egg white. The function of this peptide family has not been identified and they are potentially antimicrobial.

**Results:**

We have confirmed that there are at least 3 forms of the gallin gene in the chicken genome in 3 separate lines of chicken, all the forms are expressed in the tubular cells of the magnum region of the oviduct, consistent with its presence in egg white. mRNA expression levels are in the order 10,000 times greater in the magnum than the shell gland. The conservation between the multiple forms of gallin in the chicken genome compared with the conservation between gallin and other avian gallin like peptides, suggests that the gene duplication has occurred relatively recently in the chicken lineage. The gallin peptide family contains a six cysteine motif (C-X_5_-C-X_3_-C-X_11_-C-X_3_-C-C) found in all defensins, and is most closely related to avian beta-defensins, although the cysteine spacing differs. Further support for the classification comes from the presence of a glycine at position 10 in the 41 amino acid peptide. Recombinant gallin inhibited the growth of *Escherischia coli (E. coli) *at a concentration of 0.25 μM confirming it as part of the antimicrobial innate immune system in avian species.

**Conclusions:**

The relatively recent evolution of multiple forms of a member of a new defensin related group of peptides that we have termed ovodefensins, may be an adaptation to increase expression or the first steps in divergent evolution of the gene in chickens. The potent antimicrobial activity of the peptide against *E. coli *increases our understanding of the antimicrobial strategies of the avian innate immune system particularly those of the egg white and the evolution of the defensin family. The potential of this peptide and others in the family can now be investigated in a number of novel antimicrobial roles.

## Background

Gallin was first noted using a proteomic approach to the analysis of chicken egg white [1]. It was named gallin because of its homology to meleagrin, a peptide previously discovered as a contaminant in a turkey (*Meleagris gallopavo*) ovomucin preparation [2] and to cygnin, discovered in the preparation of black swan (*Cygnus atratus*) lysozyme [3]. Recently two similar peptides named BPS1 and 2 were found in duck (*Anas platyrhynchos*) egg white [4]. Clearly cygnin, meleagrin, gallin and BPS 1 and 2 are all peptides found in egg white and because of the large number of antimicrobial peptides already known in egg white it seems tenable that prevention of bacterial growth is a potential role for these new molecules [5]. It was speculated that meleagrin might be antimicrobial, but no evidence was observed against an *E. coli *strain [2] and no clues as to its evolution were offered, although similarities to ovotransferrin were suggested [3]. These were echoed in the examination of meleagrin [2] and the duck sequences [4]. This was based on the presence of 3 cysteines in ovotransferrin in the region proposed as homologous. One group of innate immune genes which has been well characterised in the chicken is the avian beta-defensins (AvBDs), with at least 14 members [6] which are found in a cluster on chromosome 3 [7]. Beta-defensins are characterised by their cationic nature and the presence of 3 disulfide bonds [8] and are part of a large family which are thought to work by interacting with the cell membrane of microbes to permeablise them [9]. Some human beta-defensins have been shown to have copy number variation which may be associated with Crohn's disease and psoriasis [10]. Antimicrobial peptides, along with other egg white proteins, have potentially important roles in protecting the egg and its contents from infection and have potential for exploitation in the control of microbial growth in novel ways [11].

Our overall aim in this study was to classify gallin and define its role and expression in the chicken. To that end we have confirmed the hypothesis that gallin is related to the beta-defensin family based on its structure and cysteine residues. We also confirmed the number of forms in the genome. Further we determined that its expression in tissues of the chicken was consistent with its presence in egg white and elaborated which forms are expressed in the oviduct. Finally we determined that, as hypothesised, gallin has antimicrobial properties.

## Methods

### Genomic localization and re-sequencing

Using the Staden alignment programme [12] a 364 bp core consensus sequence was determined using the following EST sequence accession numbers; EMBL: BX266328, BX266329, BX275032, BX275033, BX275163, BX275164, DT659917.

The sequence was used to perform a BLAT search [13] of the chicken genome May 2006 release using the default parameters at the UCSC genome browser [14]. Chromosome and positions on the chromosome of homologous sequence were recorded.

Primers used for re-sequencing were designed to be specific for the genomic DNA surrounding the 3 forms that were identified in the genome using the BLAT search of the chicken genome. This was achieved by masking the conserved regions using Primer 3 software [15,16]. In the case of what we termed form 1, which had a larger intron between the 2 predicted exons, 2 specific pairs of primers were used, one for each exon. The primers designed (Table 1) amplified 564 bp containing exon 1 of form 1; 657 bp containing exon 2 of form 1; 806 bp containing exon 1 and 2 of form 2; 751 bp containing exon 1 and 2 of form 3. Re-sequencing was initially carried out using 8 pure line Rhode Island red sires and subsequently 8 sires from a broiler line and 8 sires from a silkie line.

**Table 1:** Names and sequences of the primer pairs used in the study

Forward primer name	Forward primer sequence	Reverse primer name	Reverse primer sequence	Position of amplified segment chr3:
**Genomic**				
Gallin#1Exon1L	GCTCACCCCCA GACTGAATA	GallinExon1R	CTCTTCAGAGGC ACGGTGTT	109,913,588- 109,914,151
Gallin#1Exon2L	CTCCAAACCAT TGGCTGACT	GallinExon2R	GGCAAAAGGTGA CTCTGAGC	109,912,734- 109,913,390
Gallin#2Exon1&2L	TCCACGTGTTC AGCTCTTTG	Gallin#2Exon1&2R	CTCTGTGCCATTC CCATTG	109,920,204- 109,921,009
Gallin1#3Exon1&2L	CGAAGTCAGTG ATTTTCTTTCG	Gallin#3Exon1&2R	GAAGGACACCAA GGCAATGT	109,923,361- 109,924,111
**cDNA**				
Gallin AllF1	CTCCAGCCTCG CTCACAC	GallinGenomic1R2	TTGAGAGGAGGG GATGACAC	
GallinF	AGGCTATGGGC TGGTCCTGAA	GallinR	TCCTCAGCCCTTA TTTCCACT	
GallinEXF	AGGCCTGCAGC TGGTCCTGAA	GallinEXR	TCCTCTGCAGTTA TTTCCACT	

^1^Primer pairs used in re-sequencing of the 3 gallin forms in the genome, for measurement of cDNA and for recombinant expression studies. All positions refer to the May 2006 chicken (*Gallus gallus*) v2.1 draft genomic sequence assembly where appropriate.

### Bioinformatic analysis

The UniProtKB/Swiss-Prot protein database was searched using PSI-blast [17] and the zebra finch DNA database (Taeniopygia_guttata-3.2.4-contigs) [18] was searched using TBLASTN to locate potential homologues using the 41 amino acid mature gallin sequence. Further searches were made with the homologues discovered. Putative peptide sequences were aligned using ClustalW [19]. Upstream promoter regions were downloaded from Ensembl Biomart [20]. Phylogenetic trees were built using peptide or DNA sequence in Mega 4.0 [21] using the neighbour joining method. The tree nodes were tested using bootstrapping with 1000 replicates.

### Animals and tissue collection

For comparison between expression levels in different parts of the oviduct, magnum, isthmus and shell gland tissue including the mucosa, muscularis and outer serosa was obtained from 11 sexually mature hens which all had fully developed reproductive organs, however the stage at which the ovum was in the oviduct varied between individuals. Additionally tissue was taken from small intestine and cloaca. After dissection tissue was frozen in liquid N_2 _and stored at -80°C. Samples weighed on average 0.12 g. In a separate study magnum tissue was obtained from hens killed with an ovum at different stages of passage through the oviduct. Magnum tissue was processed from hens that had been killed when there was either an egg in the magnum (n = 7), an egg in the shell gland (n = 8) or when there was no evidence of ovulation that day, a so called pause day (n = 8). After dissection tissue was stored in RNA later^® ^(Ambion, Applied Biosystems, Warrington, UK) and subsequently stored at -80°C. Tissues for immunolocalisation studies (magnum, isthmus, shell gland and caecum) were harvested from 4 of these laying hens at post mortem and fixed in 10% buffered-neutral formalin (BNF) for 24 hours prior to being processed to paraffin wax using a 16 hour processing cycle on a Thermoshandon Excelsior tissue processor. All animals were killed according to schedule 1 of the animals (scientific procedures) act 1986, UK under project licence PPL 60/3964.

### RNA preparation

The tissue was homogenised in Lysing Matrix D tubes (Q-biogene-Alexis Ltd., Nottingham, UK) containing Ultraspec II total RNA isolation reagent (AMS Biotechnology, Oxon, UK) using a FastPrep FP120 homogeniser and processed as per protocol (Q-biogene-Alexis Ltd).

### Reverse transcription quantitative polymerase chain reaction (RT-QPCR) assay for all gallin forms

A 1 *μ*g sample of total RNA was reverse transcribed using a First Strand synthesis kit (GE Healthcare Life Sciences, Buckinghamshire, UK) according to the manufacturer's protocol. Reverse transcribed samples were diluted prior to use by a factor of 10 with dH_2_0. Primers Gallin AllF1 and Gallin Genomic1R2 (Table 1) were designed to amplify all forms of gallin. The programme Primer3 [15,16] was used to design the primers along with visual inspection to ensure they would amplify all genes. Reverse transcription quantitative polymerase chain reaction (RT-QPCR) was carried out using 10 μl of the diluted cDNA according to Platinum SYBR Green qPCR Supermix-UDG master mix (Invitrogen) instructions with a primer concentration of 20 mM. RT-QPCR reactions were run on an MX3000 (Stratagene) using the following conditions 95°C for 2 min, then 40 cycles of 95°C for 15 s, 60°C for 30 s. Reactions with no template were run as controls. PCR products for each amplicon were obtained using standard PCR conditions. These were purified and quantified using a Nanodrop™ spectrophotometer (Thermo Scientific) and used to construct standard curves for the determination of relative concentrations. Standards were diluted to produce top standards which were detectable during RT-QPCR amplification at around 15 cycles with six ten-fold serial dilutions forming the standard curve. Agarose gels were run to confirm that only product of the correct length free from primer-dimer were amplified by each primer pair and the product was sequenced directly. Concentrations were normalized using GAPDH measured in the same way [22]. One way ANOVA was used for statistical analysis of data using Genstat 10th edition (VSN International Ltd, Oxon, UK). Log transformation was used to give approximate normality and consistency of variances.

### Estimation of proportional expression of gallin isoforms

Re-sequencing of a layer strain of chicken indicated that the restriction enzyme digestion of the PCR product using *Nla*III and *Aci*I each distinguish one of the forms from the other two. By estimating the proportion of a single form against the other two forms it was possible to calculate the proportions of all of the forms expressed in any tissue of the layers. Form 1 amplified with Primers Gallin AllF1 and Gallin Genomic1R2 and cut with *Aci*I produces restriction fragments of 263, 59 and 2 bp whilst form 2 and 3 produced restriction fragments of 177, 86, 59 and 2 bp. Form 1 and 3 amplified with Primers Gallin AllF1 and Gallin Genomic1R2 cut with *Nla*III produces restriction fragments of 254, 39 and 31 bp whilst form 2 produces restriction fragments of 285 and 39 bp. The intensity of the bands were measured using a G:BOX imaging system (SYNGENE, Cambridge, UK) and were quantified using the gel macro facility in Scion Image Beta 4.0.3 (Scion Corporation, Frederick, MD).

### Production and titres of polyclonal anti-gallin antibodies

Two rabbits (R110 and R111) were immunized four times at three week intervals by intramuscular injection of 500 μg of folded synthesized gallin emulsified in 50% complete Freund's adjuvant for the first injection and in 50% incomplete Freund's adjuvant for the others. Rabbits were euthanized three weeks after the last injection and blood was collected by allowing it to clot at room temperature for 2 hours then storing it overnight at 4°C. Blood was centrifuged at 2000 *g *to remove blood cells and the antisera were collected and stored at -20°C.

To measure the titres of anti-gallin in the R110 and R111 antisera, synthesized gallin was diluted with 0.1 M sodium carbonate/bicarbonate buffer (pH 9.7) to a concentration of 10 μg/ml and 100 μl of the solution were added to each well of a 96-well plate. The plate was covered and stored overnight at room temperature. The wells were washed three times with phosphate-buffered saline (pH 7.4), 0.1% Tween 20 (PBST), and the plates were incubated for 90 min at 37°C with PBST, 1% Bovine serum albumin (BSA) (Sigma), to block unsaturated binding sites. Then pre-immune (null) sera and antisera were diluted 1/15 to 1/16000 with PBST, 0.2% BSA, pH 7.4. To each well, 100 μl of diluted null sera or antisera were added and the plate incubated for 1 h at 37°C. The plate was again washed three times with PBST. Goat anti-rabbit immunoglobulin-G, F(ab')2 fragment specific, conjugated to horseradish peroxidase (IgG-HRP) (Jackson ImmunoResearch Laboratories, West Groove, PA) diluted 1/625 to 1/5000 with PBST, 0.2% BSA, pH 7.4. 100 μl was applied to each well, and the plate incubated at 37°C for 1 hour. After five washings, peroxidase activity was detected by adding 100 μl/well of ABTS [2,2'azinobis(3-ethylbenzthiazolinesulfonic acid)] ready-to-use solution (Roche Diagnostics, Mannheim, Germany). After incubation for 5 to 30 min at room temperature, the absorbance at 405 nm was measured with a spectrophotometer. All animal handling protocols were carried out in accordance with the European Communities Council Directives of 24 November 1986 (86/609/EEC) and the French decree 87848 of 19 October 1987 (revised on the 31^th ^of May, 2001).

### Immunohistochemistry (IHC)

Wax embedded tissues were sectioned at 3 microns using a thermoshandon finesse microtome, lifted onto vetabond slides and incubated at 60°C for 1 hour before de-waxing and taken down to water. Each section was then treated with Proteinase K for 15 minutes at room temperature (antigen retrieval) before being loaded on to a Dako Autostainer. A standard IHC protocol was then used with optimal staining achieved using a 1:5000 dilution of the polyclonal anti-gallin antiserum (R110) for 60 minutes. The sections were viewed using a Leica DM 4000 B microscope and images captured using a Leica DC480 camera with Qwin program for PC.

### Peptide expression

A DNA fragment encoding the gallin peptide coding region was amplified by PCR using primers GallinF and GallinR from magnum cDNA. The product from the PCR was excised from an agarose gel and used in a 2^nd ^round of PCR using primers GallinEXF and GallinEXR which were partially homologous to GallinF and GallinR with the exception that a *Pst*I site was introduced. The 2^nd ^round PCR products containing the gallin coding sequence flanked by the *Pst*I restriction sites were digested with *Pst*I, purified and then ligated into the *Pst*I site of the pRSET C expression vector (Invitrogen). The recombinant vector was transformed into TOP10F' bacteria, plasmid purified and sequenced to confirm it was in the correct orientation and translational frame. Once confirmed the recombinant construct pRSET C_gallin was transformed into *E. coli *BL21(DE3-)pLysS. For expression the transformed cells were grown at 16°C in 250 ml of SOB medium. Induction of expression of the peptide was initiated by adding 1 mM of IPTG (isopropyl-*β*-D-thiogalactopyranoside) to the medium when the cell density reached 0.5 (OD_600_). The cells were cultured at 16°C for 3 hours and harvested by centrifugation at 4000 g, 4°C, for 10 min. The pellet was washed and re-suspended in 5 ml of 20 mM phosphate-buffer (pH 7.0) and lysed by sonication. The soluble fraction was recovered and proteins analysed on bisTris Mini Gels using the XCell SureLock™ system (Invitrogen) and stained with SimplyBlue™ SafeStain (Invitrogen). As a control, the peptide produced from non-recombinant pRSET C was produced in the same manner.

### Western analyses for His Tag fusion peptide expression

Soluble fractions were run as above and transferred onto a polyvinylidene difluoride membrane (Immobilon™-P; Millipore). Western blot analysis was performed using a SuperSignal^® ^West HisProbe™ Kit (Pierce, Rockford IL) as per protocol. Finally the chemiluminescence detection reaction was performed by using equal volumes of Luminol/Enhancer solution with stable Peroxide Solution (Thermo, USA), and the membrane was exposed to X-ray film for 30 seconds.

### Purification and concentration of the fusion peptides

The His-tagged fusion peptides were purified using Immobilized metal affinity chromatography (IMAC) HisPur purification cartridges kit (ThermoFisher Scientific, Perbio science, Cramlington, UK), according to the manufacturer's instructions. Briefly, 5 ml of supernatant containing gallin peptide was denatured with 5 ml of 8 M urea and incubated for 30 minutes at 4°C in a Hispur Cobalt Spin Column on an end-over-end rocking platform. The column was washed with two bed volumes of buffer 1 (100 mM NaH_2_PO_4_, 150 mM NaCl, 8 M urea, 20 mM imidazole, pH 8.0) and six bed volumes of buffer 2 (50 mM NaH_2_PO4, 500 mM NaCl, 20 mM imidazole, pH8.0) to refold and remove contaminating proteins whilst still bound to the column. The recombinant His-tagged fusion peptides were then eluted with 9 ml of 50 mM elution buffer (50 mM NaH_2_PO_4_, 500 mM NaCl, 250 mM imidazole pH8.0). The fusion proteins were concentrated using centrifugal filters with a molecular weight cut off of 3 kDa (Millipore, Carrigtwohill, Co. Cork, Ireland). This was passed through the filter 3 times with 20 mM phosphate buffer in order to transfer the peptide into this buffer. Protein concentration was determined by a Coomassie Plus (Bradford) Assay Kit (Pierce) using bovine serum albumin as the protein standard.

### Antimicrobial assay

The antimicrobial assay method was essentially as described previously [23,24]. *E. coli *BL21(DE3-)pLysS was cultured at 37°C overnight in Luria broth (LB). Two hundred and fifty μl of overnight culture was sub-cultured into 20 ml of LB and incubated for 3 hours at 37°C. After the second incubation, 20 μl of culture was diluted with 2 ml of PBS. Ten microliters of gallin (1.5, 3.0, 6.0 μM) or pRSETC control peptide extract (1.5, 3.0, 6.0 μM) or PBS (control) was added to 50 μl of diluted culture to produce final concentrations of 0.25, 0.5 and 1.0 μM respectively. This was vortexed and incubated for 3 h at 37°C and then the suspensions were serially diluted to 1 × 10^-4 ^with PBS, the 1 × 10^-3 ^and 1 × 10^-4 ^dilutions being plated on agar plates. All plates were incubated overnight at 37°C and the colonies were counted. Ten microliters of ampicillin (300 μg/ml) were used as a positive control.

## Results

### Genomic location and bioinformatics

**Figure 1:**
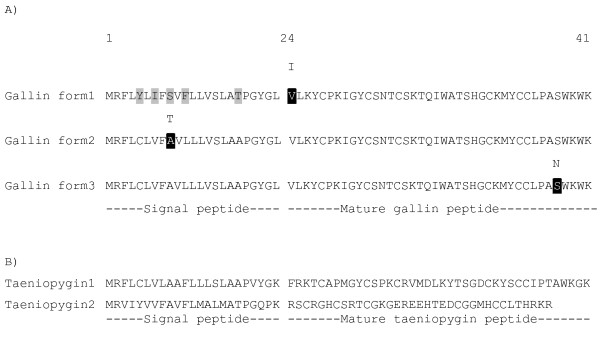
**A) Alignment of the 3 gallin predicted peptide forms**. The sequences are referenced to the layer line sequence. Predicted polymorphism in the peptide sequence observed from sequencing genomic DNA from lines of layer, broilers and silkie hens (n = 8/line) are shaded in black with white type with the alternative amino acid shown above the sequence. Differences between the 3 sequences are indicated by light shading and are found only in the signal peptide. B) Predicted protein sequence of ovodefensins from zebra finch (*Taeniopygia guttata*). Taeniopygin 1 is homologous to gallin and taeniopygin 2 to duck BPS1.

The 364 bp EST consensus sequence containing the predicted coding region of the gene had high identity to 3 locations in the May 2006 chicken (*Gallus gallus*) v2.1 draft genomic sequence assembly; 99.2% to a region on the minus strand of chromosome 3 (109912867-109913892) spanning 1026 bp (form 1); 97.8% to a region on the minus strand of chromosome 3 (109920354-109920879) spanning 526 bp (form 2); 96.7% to a region on the positive strand of chromosome 3 (109923486-109924011) spanning 526 bp (form 3). When the regions containing the sequences with high similarity to the core consensus sequence were re-sequenced the result obtained was 99.7% identical to the May 2006 chicken (*Gallus gallus*) v2.1 draft genomic sequence assembly for form 1 with 1 difference in an exon which is the site of a known SNP (snp.17.145.10816.S.2) and a 4 base deletion which restores a putative TATAA site; 99.6% identical for form 2 including a known SNP (snp.17.145.2833.S.1) but in the case of form 3 the identity was only 98.7% due to some mismatches near the site of a 10 bp gap in the genome assembly. The result of re-sequencing the 3 forms of gallin for all 3 strains of chicken have been submitted to the EMBL nucleotide database with accession numbers FN550404-FN550415. The sequencing of the third form also demonstrated that there was not a premature stop codon in the sequence (accession number FN550413-FN550415. This had been suggested by the May 2006 chicken (*Gallus gallus*) v2.1 draft genomic sequence assembly but it is at a site in the v2.1 draft genomic sequence where two contigs (contig 17.145 and 17.144) abut and this is likely to explain the discrepancy. Re-sequencing of 24 sires from 3 lines of chicken (Broiler, layer and silkie) using primers unique for the genomic DNA flanking the 3 forms of gallin demonstrate that each is present in the genome of all lines and that each gene locus is unique. In the sequence obtained from the laying strain all the predicted peptide sequences from the 3 forms were identical, except in the signal peptide (Figure 1A). If the 23 amino acid signal peptide is included form 2 and form 3 have identical protein sequences whilst form 1 is 92% identical and 95% similar to form 2 and form 3. However, there were some non-synonymous polymorphisms observed in broilers and silkies in addition to the allelic variant seen in the layer strains which caused conservative changes to the amino acid sequence (Figure 1A). These were I24V in gallin form 1, T9A in gallin form 2 and N59S in gallin form 3.

The mature gallin peptide of 41 amino acids shares 62% identity and 72% similarity with cygnin and 65% identity and 83% similarity with meleagrin. In addition to gallin, meleagrin and cygnin a further protein, BPS2, was identified in duck egg white that had an identical sequence to cygnin with accession number Swiss-Prot: P85124[4] and a putative peptide in the zebra finch from the Jul. 2008 *Taeniopygia guttata *draft assembly at position 111,191,062-111,191,184 on chromosome 3 with an identical copy at 111,176,525-111,176,647 (Figure 1B). A further potential paralog in the zebra finch was found at chr3:111,194,305-111,194,418 (Figure 1B) which may be homologous to BPS1, Swiss-Prot P85123[4]. We named these taeniopygin1 and 2 (Figure 1B).

**Figure 2:**
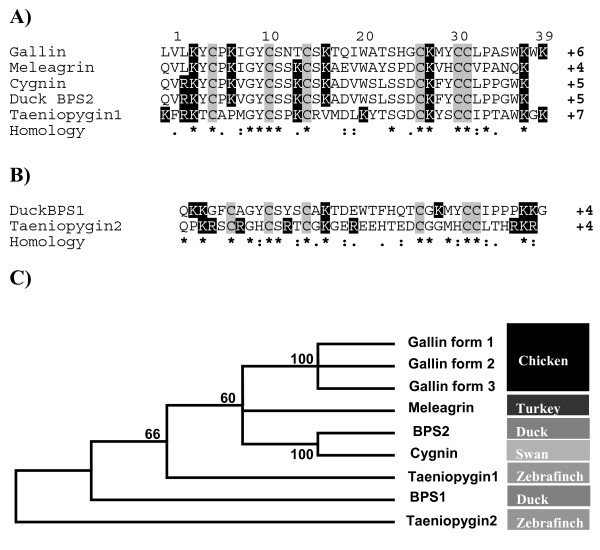
**The ovodefensin family**. A) CLUSTALW 2.0.11 multiple sequence alignment of ovodefensins. Only the mature peptides are used. Conserved cysteines are shown with a light shaded background and cationic residues with a black background and white type. Complete conservation is indicated with '*', similarity with '**:'**, and weakly similar with '**.'**. The relative charge is indicated at the end of each sequence. Where the Latin name has been used as the basis for the peptide name the common name for the species can be found in part C adjacent to the peptide names. B) Aligned sequence of two peptides closely related to the ovodefensins found in duck and zebra finch. Legend as A. C) A phylogram indicating the evolutionary history of the ovodefensins was inferred using the Neighbor-Joining method [41] from an alignment of the mature peptides shown in A and B. The tree is the consensus of 1000 replicates with the percentage of replicate trees in which the branches clustered together in the bootstrap test shown next to the branches. Branches corresponding to partitions reproduced in less than 50% of bootstrap replicates are collapsed. All positions containing alignment gaps and missing data were eliminated only in pairwise sequence comparisons. The common name for the species where the peptides have been isolated can be found to the right of the peptide names. We can see that the 3 molecules identified in the chicken are more similar to each other than the molecules from the other species.

The gallin, cygnin, meleagrin, taeniopygin 1 and duck BPS2 mature peptides contain six cysteines spaced in a C-X_5_-C-X_3_-C-X_11_-C-X_3_-C-C motif at position 6, 12, 16, 28 and 32-33 in the respective mature peptides (Figure 2A). All are relatively cationic with a number of conserved arginine residues (Figure 2A). Within the ovodefensins there are 2 groups, those containing gallin, meleagrin cygnin, BPS2 and taeniopygin1 (Figure 2A) and a second group which contains taeniopygin2 and duck BPS1 (Figure 2B). This second group has a C-X_3_-C-X_3_-C-X_11_-C-X_4_-C-C motif however in the phylogeny presented only taeniopygin2 appears as an outgroup (Figure 2C).

**Figure 3:**
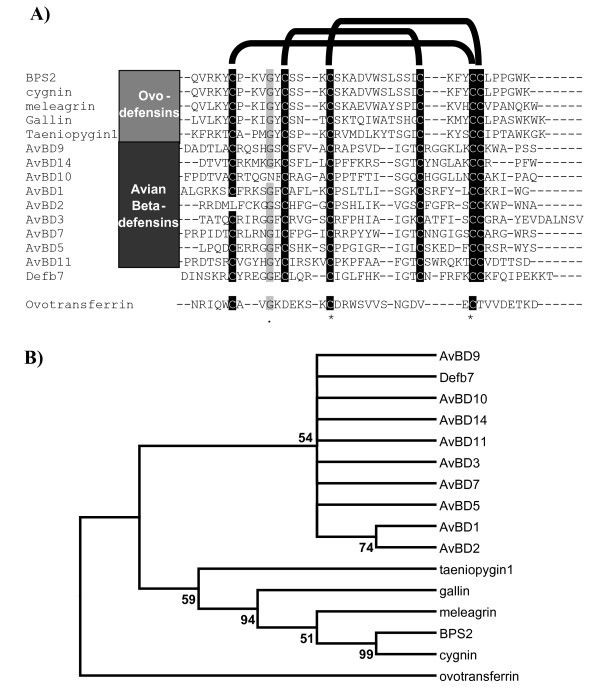
**Comparison of ovodefensins, avian beta-defensins from chicken and chicken ovotransferrin**. A) CLUSTALW 2.0.11 multiple sequence alignment of ovodefensins (top), avian beta-defensins (middle) and the ovotransferrin sequence (bottom) used by Odani [2]. The shading shows greater than 85% identity and features the conserved cysteines and the glycine at position 10 of the ovodefensin peptides. Complete conservation between all molecules is indicated with '*' and weakly similar with '**.'**. The black bars at the top of the diagram indicate the known cysteine bonds in avian beta-defensins and ovodefensins. The avian beta-defensins nomenclature follows that suggested in [6]. A mouse defensin, mouse beta-defensin 7, NP_631966 (Def7), was included to indicate the relationship between avian beta-defensins, mammalian defensins and ovodefensins. Where the Latin name has been used as the basis for the peptide name the common name for the species can be found in Figure 2C adjacent to the peptide names. B) The evolutionary history was inferred from the alignment in A using the Neighbor-Joining method as detailed in figure 2 with the exception the complete deletion option was used.

Comparison of alignments of ovodefensins with known AvBDs indicates that the cysteine arrangement is conserved although the spacing between the cysteines differs from one to three amino acids (Figure 3A). The phylogeny including ovotransferrin and a mouse beta-defensin (Defb7) shows that the ovodefensin molecules form their own group. The branch lengths are shorter (Figure 3B) indicating that the ovodefensins appear to be more conserved than the AvBDs. The molecules are more similar to beta-defensins than to ovotransferrin (Figure 3B) which appears as an outlier.

**Figure 4:**
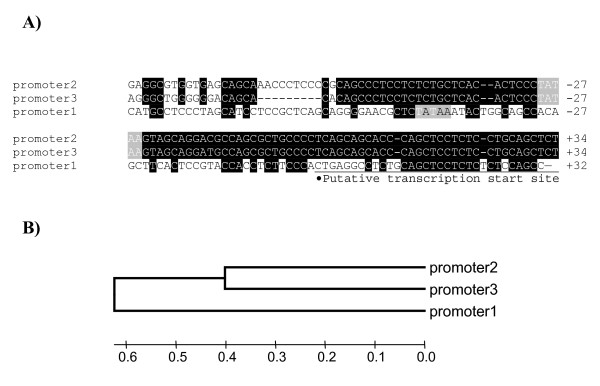
**The gallin promoter**. A) CLUSTALW 2.0.11 multiple sequence alignment of the promoter region of the 3 forms of gallin. Black background and white letters indicates complete conservation between form 2 and 3 which extends ~60 bp upstream, and where appropriate conservation with form 1 is similarly indicated. Light shading indicates putative TATAA boxes, the transcription start site is indicated with a triangle and the first exon is underlined. B) The evolutionary history was inferred using the Neighbor-Joining method [41]. The phylogenetic tree was linearized assuming equal evolutionary rates in all branches [42]. The tree is drawn to scale, with branch lengths in the same units as those of the evolutionary distances used to infer the phylogenetic tree. The evolutionary distances were computed using the Maximum Composite Likelihood method [43] and are in the units of the number of base substitutions per site. All positions containing gaps and missing data were eliminated from the dataset (Complete deletion option). There were a total of 489 positions in the final dataset.

The analysis of the promoter region indicates that the proximal promoter in form 2 and form 3 are 95% identical for 60 bp upstream of the putative transcription start site, thereafter they are about 38% identical over 448 bases. All forms share 38% identity in the 60 bp upstream of the putative transcription start site and about 32% over 448 bases upstream (Figure 4).

### Tissue expression

**Figure 5:**
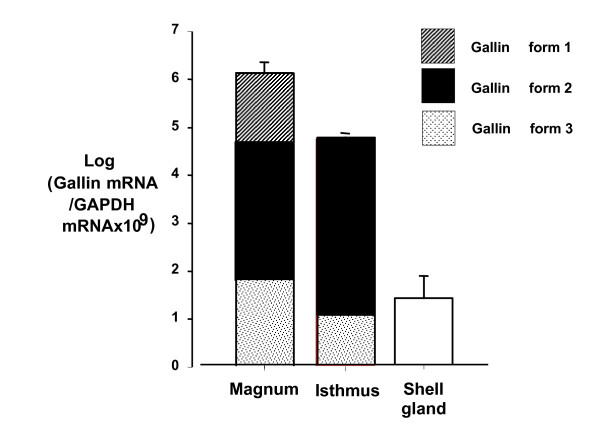
**Expression of gallin mRNA in hen oviduct tissue; magnum, isthmus and shell gland measured by RT-QPCR (n = 8, mean ± sem)**. The expression is corrected for GAPDH expression to attempt to normalise for any differences in tissue sample size and is presented on a logarithmic scale because of the large difference in expression between shell gland and magnum. The different shading for the magnum and isthmus indicate the proportion of expression of the 3 forms of gallin (indicated in the inset key). Note that the proportions of the 3 forms are indicated on the arithmetic scale. Differences between all tissues are significant at P < 0.001.

**Figure 6:**
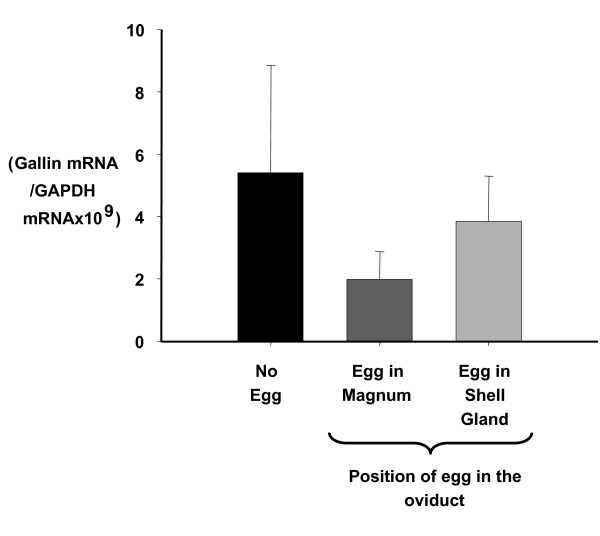
**Expression of gallin in the magnum of the hen oviduct measured by RT-QPCR (n = 8, mean ± sem)**. The expression is corrected for GAPDH expression to attempt to normalise for any differences in tissue sample size. 'No egg' represents a pause day when the hen did not ovulate.

Measurement of gallin expression indicated that the amount of gallin mRNA in the magnum of the oviduct was around 100 times more than in the isthmus, which was in turn around 140 times more than in the shell gland (Figure 5). Levels in small intestine and skin were even lower than in the shell gland (data not shown). Using specific restriction digests the proportions of the 3 forms (mean ± sem) of the mRNA in the magnum was: form 1, 26.8 ± 2.8%; form 2, 52.1 ± 3.2%; form 3, 25.4 ± 3.0% and in the isthmus: form 2, 85.8 ± 5.4%; form 3, 14.2 ± 5.4% and form 1 was not expressed (Figure 5). Expression in the magnum did not differ significantly whether the egg was in the magnum or in the shell gland or if it was a pause day (Figure 6).

### Production and titres of polyclonal anti-gallin antibodies

Two rabbits were immunized against synthesized gallin to produce polyclonal IgG antibodies against gallin. The ELISA determined titres of each antiserum following the first immunisation showed high cross reactivity determined by colour production when compared to pre-immune serum. At an antiserum dilution of 1/156, values were five times that of pre-immune serum. With the third immunisation, values increased further and at this level of antibody production, values at 1/156 dilution were eight times the value for pre-immune serum.

### Immunohistochemistry

**Figure 7:**
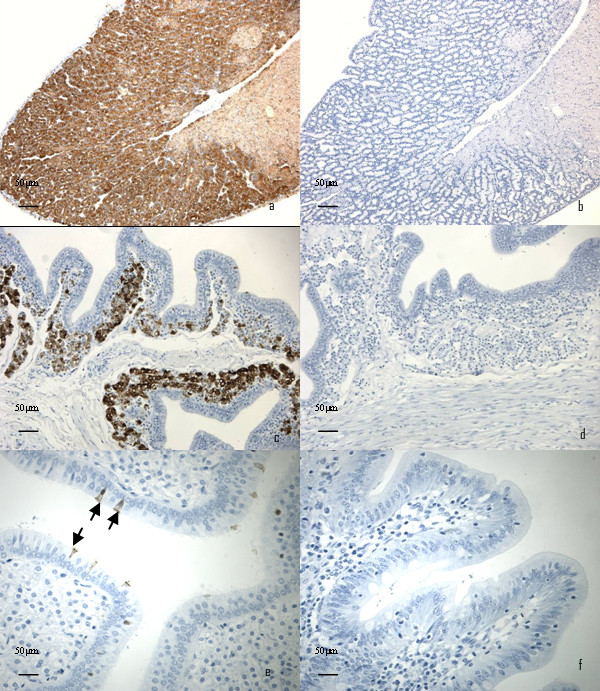
**The tubular gland cells of the magnum and shell gland region of the oviduct stained positive with anti-gallin antisera (R110) when the egg was in the shell gland region (a and c)**. Corresponding negative controls are shown in images b and d. Positive staining was limited to a few of the ciliated epithelial cells lining the lumen of the shell gland when the egg was more proximally placed in the oviduct (e). The caecum was not reactive to the primary antibody (f).

The anti-gallin antiserum (R110) obtained as described above, produced positive staining in the tubular glands of both the magnum and the shell gland (Figure 7a and 7c). The variation in staining density observed in figure 7a is due to regional differences in the secretory activity of the tubular gland cells. The staining activity of the tubular gland cells in the shell gland was highly dependant on the stage of egg formation. (Figure 7c and 7e). A proportion of the ciliated cells associated with the surface epithelium in both the magnum and shell gland (Figure 7) also stained positive, irrespective of the phase of the laying cycle (Figure 7e arrows). No staining was observed in caecum (Figure 7f) and no staining was observed in any of the tissues in the absence of the primary antibody (Figure 7b, d).

### Antimicrobial activity

**Figure 8:**
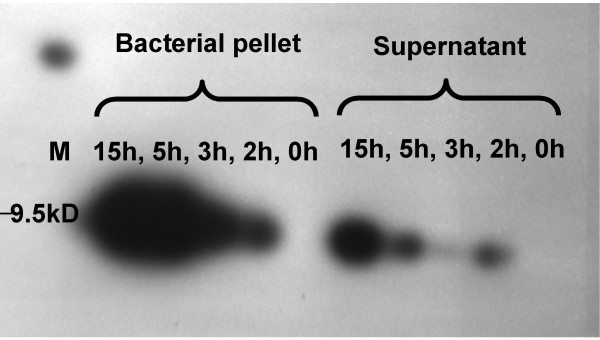
**Recombinant gallin identified by western blotting performed using a SuperSignal^® ^West HisProbe™ Kit (Pierce, Rockford IL and visualised with Luminol/Enhancer solution with stable Peroxide Solution (Thermo, USA)**. The membrane was exposed to X-ray film for 30 seconds. The lanes show the signal from samples derived from either the supernatant or the bacterial pellet after induction by 1 mM IPTG for 0, 2, 3 5 or 15 hours. Molecular weight was determined by interpolation from a Spectra™ Multicolor Broad Range Protein Ladder (Fermentas) run in lane M.

**Figure 9:**
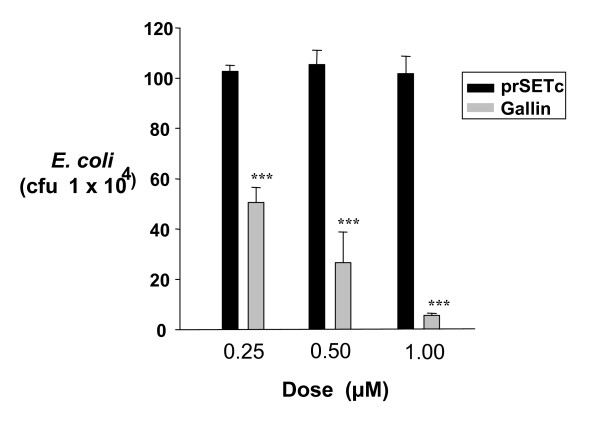
**Recombinant gallin or control peptide at 0.25-1.0 μM was incubated for 3 h at 37°C with *E. coli *BL21(DE3-)pLysS in PBS and the number of surviving bacteria counted**. *** = P < 0.001.

Recombinant peptide was detected in western blots of His Tag purified protein migrating at the anticipated size of around 9.5 kilo Daltons after IPTG induction (Figure 8). Purification was confirmed by increased signal using the HisProbe antibody after concentration. Purified preparations of recombinant protein containing the gallin peptide showed a relatively dramatic effect on the survival of *E. coli *with around 50% inhibition demonstrated at 0.25 μM and 95% inhibition at 1 μM (Figure 9). The control peptide showed no inhibition of bacterial growth at these concentrations (Figure 9).

## Discussion

We have collected a number of strands of evidence which support our hypothesis that gallin is a member of a new class of antimicrobial peptides found in egg white, the ovodefensins, which are related to the beta-defensins.

Simpson [3] followed by Odani [2] compared the cygnin and meleagrin sequences to part of avian ovotransferrin. The alignment between the 2 proteins alone, meleagrin and ovotransferrin, suggested conservation of 7 residues out of 40 with only half of the key cysteine residues conserved. In the egg white proteome paper of Mann [1] no suggestion was made to the nature of the gallin like peptides and Nanukool et al. [4] followed the lead of Odani. However the gallin, cygnin, meleagrin, taeniopygin1 and duck BPS2 mature peptides all have a C-X_5_-C-X_3_-C-X_11_-C-X_3_-C-C motif (Figure 2A, 3). Comparison of alignments with known AvBDs indicates that the cysteine arrangement is conserved although the spacing between the cysteines differs (Figure 3A). Seven residues (6 cysteines and one glycine) are almost completely conserved between the ovodefensins and the AvBDs (Figure 3A). But only 2 residues are conserved if ovotransferrin is included in the alignment (Figure 3A). The general sequence of beta-defensins is C-X_6_-C-X_4_-C-X_9_-C-X_6_-C-C [25] but a more relaxed consensus is C-X_(4 to 8)_-C-X_(3 to 5_-C-X_(9 to 13)_-C-X_(4 to 7)_-C-C [7] so the ovodefensins have a shorter spacing between the 4^th ^and 5^th ^cysteines. There is some variation in the spacing within the different defensin families, although none have this pattern or such a short distance between the 4^th ^and 5^th ^cysteine [7]. It appears that the position expected for the fourth cysteine, which would be at position 25 in the gallin like ovodefensins, is substituted with a serine however there is a conserved cysteine at position 28 (Figure 2A and 3) in the gallin like ovodefensins. Other defensins such as the alpha family have a cysteine spacing [9] which is even further from the ovodefensins than the beta-defensins. Determination of the cysteine bonds in meleagrin and the duck BPS1 and BPS2 peptides [2,4] suggests that the bonds between cysteines (Figure 3) are also conserved between defensins and the ovodefensin molecules, further support for the view that these peptides are related to beta-defensins. Overall the peptide is relatively serine rich with 3 sets of conserved serines at 13, 17 and 25 (Figure 2A) although this is not unusual in other AvBDs (Figure 3).

We would argue that the location on chromosome 3, close to the beta-defensins [7], the conservation of the positions of the cysteine with the beta-defensin family (Figure 3), and distinctive motif of 6 spaced cysteines suggests the family are related to the defensins and possibly most closely to the avian beta-defensins. Furthermore there is conservation of the glycine at position 10 in the mature peptide (Figure 2A and 3) consistent with the beta-defensin family [25,26] and the serine at position 13 is also relatively well conserved. The peptides are also relatively short with the cysteine containing motif being immediately after the signal peptide similar to many of the beta-defensins (Figure 3) but dissimilar to mature ovotransferrin which is 686 amino acids in length [27]. Therefore we propose that these molecules are not related to ovotransferrin and are a new family of antimicrobial peptides, the ovodefensins, related to the beta-defensins.

The presence of potential homologs in duck and zebra finch suggest that the peptides are present across all the avian vertebrate classes from passerines to anseriformes (Figure 2A, C) and there may be further diversification of the family with potential related peptides being identified in the duck (BPS1) [4] and in the zebra finch (taeniopygin2) which have a spacing of C-X_3_-C-X_3_-C-X_11_-C-X_4_-C-C (Figure 2B). In the phylogeny however only taeniopygin2 appears as an outgroup (Figure 2C), this is due to the relatively high conservation between BPS1 and the ovodefensins outside the key cysteine, arginine and serine residues and the spacing imposed in the alignment.

The inclusion in the phylogeny (Figure 3A) of a mouse defensin which groups within the defensins indicates that although gallin is related to the defensins it is possible that the ovodefensins have diverged before the avian beta-defensin family separated. In these alignments considerable gaps need to be introduced to make the alignment because of the different cysteine spacing but overall it suggests that the peptides are a separate group from the avian beta-defensins. However, in the absence of identifiable ovodefensins in other taxa it makes it difficult to establish the exact relationship. Although they may simply be undetected, it seems possible that the ovodefensins are specific to birds as no similar peptide was observed in the lizard (*anolis carolinensis*) genome or other in any other genome. This might be because either the genes were lost due to a move to viviparity, which the lack so far of the gene in lizards would gravitate against, or they have evolved only in the avian lineage.

Examination of the chicken genome [28] indicated that there were three potential forms of the gene encoding gallin on chromosome 3. One form apparently contained a premature stop codon but we have demonstrated that it is a sequencing artefact in the genome build. Therefore all forms can potentially transcribe full length peptides. The presence of three forms in the genome, which we have confirmed by sequencing in three lines of chicken, suggests that this peptide may have been duplicated to increase production of the protein for inclusion in egg white since all three forms appear to be expressed in the magnum (Figure 5). Two of the forms, form 2 and form 3, have similar proximate promoters (Figure 4) and were expressed outside the magnum in the isthmus in moderate quantities unlike form 1 which was only observed in the magnum (Figure 5). Although some conservative substitution was observed in the sequence of gallin in silkie and broiler lines (Figure 1A) it still remains that the three forms are almost identical and produce almost identical peptides (Figure 1A). Because these forms are more similar to each other than they are to any of the other family members such as meleagrin or cygnin it is possible that these duplications are very recent in the chicken lineage. In other words gallin may be like avidin, another egg white gene involved in innate immunity, where there is more than one copy [29]. Furthermore, like avidin and some beta-defensins [30] the possibility might exist that the number of copies may vary within an individual but we have observed no evidence in this study from the sequencing results. However this would not be picked up if the entire region was duplicated. It is of course also possible that multiple copies of ovodefensins are present in other species. We know that 2 peptides are expressed in the duck oviduct since the reported sequences BPS1 and 2 were found in egg white [4] and two putative forms are present in the zebra finch genome which appear identical and a further sequence which is similar (Figure 1B). The sequences in duck are clearly different and seem to represent a different evolutionary form which is similar to taeniopygin2. It is possible that the two identical zebra finch sequences may be an assembly artefact since the chicken sequence was used as a scaffold because of the similarity between the genomes [31]. Either way, we have clear evidence that there has been recent duplication in the chicken genome of the gallin molecule. This may be similar to observations of species specific multiplication of the murine β-defensin locus, although greater diversification has occurred in these genes than in the case of gallin [32].

All the available evidence suggestes that the ovodefensins are present in egg white [1-4] being about the 18th most abundant peptide as revealed by the protein abundance index in mass spectrometry [1]. Therefore gallin would be expected to be highly expressed in the magnum of the oviduct where egg white is synthesised. This was true, with all forms being expressed in that tissue (Figure 5). Using primers which detected all forms we estimated that the expression level was over 4 orders of magnitude greater in the magnum than the shell gland of the oviduct. The isthmus, which is adjacent to the magnum and is thought primarily to produce the eggshell membrane, had around 40 times less expression of gallin than the magnum (Figure 5). Expression in other tissues where antimicrobial peptides are also known to be expressed such as the small intestine or cloaca were lower and essentially undetectable (data not shown). Immunohistochemical localisation demonstrated that the most intense staining was in the tubular gland cells of the magnum as expected from its discovery in egg white (Figure 7a), however there was also positive staining in the tubular gland cells of the shell gland, suggesting that although expression was lower it may still play a role as an antimicrobial in this region of the oviduct (Figure 7c). The secretory activity in the shell gland however seems to be highly dependant on the stage of the laying cycle with little or no activity being detected when the egg is proximally situated. No staining was seen in the caecum of the intestine (Figure 7f) reinforcing the RT-QPCR results.

The proportions of the 3 forms of the mRNA in the magnum were dominated by form 2 but that all forms were expressed (Figure 5). In relation to the expression in the isthmus it would appear that form 1 is not expressed in this tissue and form 2 predominates. Form 1 may be the original form for expression in egg white and the extra forms seem to be slightly less precise in their expression possibly because of the differences in the proximal promoter sequence. The analysis of the promoter region indicates form 2 and form 3 are almost identical 60 bp upstream of the putative transcription start site. Thereafter their similarity is around 32% between all the forms (Figure 4). This suggests that form 2 and form 3 are more similar and it is of note they are expressed principally in the isthmus. It is also interesting to note that a sequence of about 70-80 bases in the promoter between about 263 and 335 bp upstream can be found represented up to 38 times on chromosome 3 and many times elsewhere in the genome which may have facilitated an increase in the rate of gene duplication or segment exchange by non-homologous recombination.

The expression pattern of mRNA for gallin during an ovulatory cycle shows no change (Figure 6). This is perhaps not surprising since the ovalbumin gene, which is the main protein in egg white, only changes slightly over a similar time span [33]. This may be because the tissue secretes a large amount of protein over a relatively short period but has to synthesise and store those proteins continuously to supply the once a day demand. In the shell gland, where large changes in expression are observed for shell organic matrix proteins, the secretion is over a much longer time span [34].

All the ovodefensins are relatively cationic with a number of conserved arginine residues (Figure 2A) which is unlike the AvBD which have few arginine residues but a greater number, frequently at least 6, of lysine. The large number of arginine residues is a feature of defensins which are found in granular structures and has been proposed to aid storage [9] which may also be important in the magnum. There are no aspartic acid or glutamic acid anionic residues in gallin and overall the net positive charge is greatest for gallin (+6) and taeniopygin1 (+7) (Figure 2A). The mechanism of action of AvBDs is not completely known, but exposed cationic sites are thought to interact electrostatically with negatively charged membrane components of bacteria [35]. Then, after peptide accumulation, parallel to the membrane surface, dimers and multimers could be formed, resulting in the creation of a pore [9,36]. A role as antimicrobial peptides would therefore appear to be a likely function for this peptide family. The fact that the egg white is known for its armoury of antimicrobial proteins and peptides [5] that protect the embryo during incubation suggests that this might be a function of this peptide and, of course, defensins are potent antimicrobials [9]. Cygnin purified from egg white was not observed to have antimicrobial activity [3], however in this study we saw clear inhibition of *E. coli *with relatively low levels of recombinant gallin (0.25 μm) (Figure 9). Using a similar preparation of beta-defensins 4, 7 and 9, inhibition of salmonella serovars was observed at 2 μM [37] and human defensin 118 showed inhibition of *E. coli *at around 1 μM [38]. This is further evidence for gallin as a member of the defensins. Defensins are know to work better at lower concentrations of NaCl [39], the concentration used in this analysis was relatively high at 170 mM suggesting the activity of gallin might be even greater at lower concentrations of NaCl. The concentration of sodium ions in egg white is around 63 mM [40].

## Conclusion

To summarise we have examined the evolution of a new family of peptides, the ovodefensins. We have examined in detail the biology of gallin, the chicken representative of this family, which is most abundantly expressed in oviduct tissues, consistent with proteomic observations. The family contains a number of features which suggests it is part of the defensin family and is probably related to the beta-defensins, perhaps having diverged from a common ancestor at the time the avian beta-defensins evolved. The categorisation of defensins is based to some extent on the cysteine spacing and this family would represent a new variation on the known beta-defensins [7]. In the chicken and possibly other avian lineages there is evidence for relatively recent duplication of the gene with a surprising level of conservation within the species. All these forms are expressed, and overall the highest expression is in the tubular secretory cells of the magnum of the oviduct as expected. Whether the recent duplication is an adaptation to increase quantities of expression or, alternatively, we are just catching a snap shot of evolution in action providing the tools for evolution of peptides with different specificity is unknown. We observed no evidence of copy number variation with all animals and lines examined containing all 3 forms but it may still exist. Lastly we have demonstrated that the peptide has potent antimicrobial activity against E. *coli *which may indicate new uses for these peptides and increases our understanding of the antimicrobial strategies of the avian innate immune system.

## Authors' contributions

DG and PWW carried out sequence and expression analysis, recombinant expression and analysis of data, MMB and KMcD carried out immunohistochemistry and sample collection. JK developed the assay for different forms. VH-G and YN developed the antibody for immunochemistry. YN, MMB and ICD gained funding. ICD carried out the bioinformatics and phylogeny analysis and conceived the project. All authors contributed and approved the final manuscript.
